# Systematic review of the diagnostic accuracy of thick smear compared to polymerase chain reaction for pregnancy-associated malaria, 2010-2022

**DOI:** 10.17843/rpmesp.2022.393.11739

**Published:** 2022-09-30

**Authors:** Roxana Gómez-Hoyos, Jaiberth Antonio Cardona-Arias, Luis Felipe Higuita Gutiérrez, Walter Salas-Zapata, Jaime Carmona-Fonseca

**Affiliations:** 1 Escuela de Microbiología, Universidad de Antioquia, Medellín, Colombia. Universidad de Antioquia Escuela de Microbiología Universidad de Antioquia Medellín Colombia; 2 Universidad de Antioquia, Medellín, Colombia. Universidad de Antioquia Universidad de Antioquia Medellín Colombia; 3 Universidad Cooperativa de Colombia, Medellín, Colombia. Universidad Cooperativa de Colombia Universidad Cooperativa de Colombia Medellín Colombia; 4 Grupo Salud y Comunidad César Uribe Piedrahíta, Facultad de Medicina, Universidad de Antioquia, Medellín, Colombia. Universidad de Antioquia Grupo Salud y Comunidad César Uribe Piedrahíta Facultad de Medicina Universidad de Antioquia Medellín Colombia

**Keywords:** Thick Blood Smears, PCR, Diagnostic Accuracy, Malaria Associated with Pregnancy, Meta-analysis

## Abstract

**Objective.:**

To evaluate the accuracy of thick smear (TS) versus quantitative polymerase chain reaction (PCR) for pregnancy-associated malaria (PAM).

**Materials and methods.:**

We carried out a systematic review of diagnostic tests in nine databases. Methodological quality was evaluated with QUADAS. Sensitivity, specificity, positive likelihood ratio (PLR), negative likelihood ratio (NLR), diagnostic odds ratio (DOR) and area under the ROC curve were estimated. Heterogeneity was determined with the Der Simonian-Laird Q method and uncertainty with the weighted percentage of each study on the overall result.

**Results.:**

We included 10 studies with 5691 pregnant women, 1415 placentas and 84 neonates. In the studies with nested PCR (nPCR) and quantitative PCR (qPCR) as the standard, the diagnostic accuracy results were statistically similar, with very low sensitivity (50 and 54%, respectively), high specificity (99% in both cases), high PLR and poor NLR. When nPCR was used, the DOR was 162 (95%CI=66-401) and the area under the ROC curve was 95%, while with qPCR it was 231 (95%CI=27-1951) and 78%, respectively.

**Conclusions.:**

We demonstrated that research on the diagnostic accuracy of TS in PAM is limited. Microscopy showed poor performance in the diagnosis of asymptomatic or low parasitemia infections, which reinforces the importance of implementing other types of techniques for the follow-up and control of malaria infections in pregnant women, in order to achieve the control and possible elimination of PAM.

## INTRODUCTION

*Plasmodium* spp. infection during pregnancy (pregnancy-associated malaria or PAM) includes three clinical pictures: gestational malaria (GM) or demonstrated infection in the mother’s peripheral blood; placental malaria (PM) or presence of *Plasmodium* spp. in the placenta, and congenital malaria (CM), which corresponds to infection of the newborn during intrauterine life or delivery, with the presence of *Plasmodium* spp. in the first seven days of life or later, with clinical manifestations between 10-30 days postpartum ^1^ ^-^ ^5^. PAM presents serious risks for the pregnant woman, such as anemia, cerebral malaria, severe malaria and death; in addition, it causes negative outcomes in the fetus and newborn such as intrauterine growth restriction, anemia, intrauterine death, premature delivery, low birth weight, among others ^1^ ^,^ ^2^.

The World Health Organization (WHO) indicates that sub-Saharan Africa has a moderate-high prevalence of PAM, estimating that out of 33.2 million pregnancies, 35% were exposed to malaria; likewise, in Central Africa there is a 40% risk of malaria transmission in pregnant women ^6^. In the Americas, 1% of malaria cases reported between 2010 and 2016 occurred in pregnant women, causing fourteen deaths in this population group ^7^.

The thick blood smear (TBS) is the most widely used diagnostic test for malaria and is the standard test in endemic areas. This can be corroborated in some clinical practice guidelines of the Pan American Health Organization and the Spanish Society of Tropical Medicine and International Health (SEMTSI) ^8^ ^,^ ^9^. However, this test causes a high underestimation of cases compared to what is found with molecular tests. For example, a meta-analysis in Colombia concluded that when using TBS for PAM detection the prevalence was 4.5% (95%CI: 2.9-6.9), for GM it was 5.8% (95%CI: 3.8-8.7), for PM it was 3.4% (95%CI: 1.7-6.7) and for CM it was 1.3% (95%CI: 0.6-3.0); while when using PCR, the prevalence of PAM was 14.4% (95%CI=7.6-25.5), for GM it was 16.7% (95%CI: 9.0-28.8), for PM it was 11.0% (95%CI: 4.1-26.3) and for CM it was 16.2% (95%CI: 8.2-29.5) ^10^. However, in Colombia, a study on malaria carried out with 314 samples found different results, reporting a sensitivity of 97% and a specificity of 100% comparing TBS performed at the microscopy stations with PCR from the reference laboratory. It is important to mention that this research was carried out in a symptomatic population that does not reflect the conditions of the general population and does not capture the entire clinical spectrum of the disease from the asymptomatic stages ^11^.

TBS has sensitivity problems when used in infections with low parasite density and in asymptomatic infections, but this is not the case for molecular methods such as PCR. PCR has been reported to be important for the detection and treatment of individuals with low parasitemia that can act as reservoirs of transmission and hinder the goals of disease elimination ^12^. Overall, TBS is considered as the reference standard for malaria; however, in cases of PAM it is more appropriate to use PCR as the standard due to the following reasons: (a) the frequency of submicroscopic, low-density and asymptomatic infections is higher in PAM, which are not captured with TBS, (b) TBS has a lower detection limit than PCR (10-30 parasites/microliter of blood, but in field conditions this figure can rise to 50-100 parasites/microliter of blood), c) TBS is suitable for clinical (symptomatic) malaria, but not for asymptomatic malaria, so it is not adequate as a standard for epidemiological surveillance programs), and d) advances in the last decade regarding molecular diagnosis are evidence of the better diagnostic capacity of PCR ^10^ ^,^ ^11^ ^,^ ^13^ ^,^ ^14^.

Despite the discrepancies between TBS and PCR in the detection of *Plasmodium* spp., no research in the scientific literature has synthesized and evaluated the available evidence on the diagnostic accuracy of TBS in gestational, placental or congenital malaria; even for other population groups, studies on the diagnostic accuracy and performance of this test are scarce. In addition, there are no systematic reviews related to the subject; there is one study that systematized the research on diagnostic accuracy of tests for pregnancy-associated malaria; however, it focused on rapid tests and included studies between 1914 and 2009 ^15^, but the main advances in molecular diagnostics have been made after 2009.

Therefore, the aim of this systematic review was to evaluate the diagnostic accuracy of TBS versus PCR for PAM, in 2010-2022. This study is relevant in order to improve clinical practices, generate better quality evidence, estimate diagnostic accuracy parameters with greater precision and possibility of extrapolation, consolidate hypotheses on the need to improve diagnostic methods, increase the accuracy of the conclusions of individual studies and identify areas of uncertainty where additional research is needed ^16^. Furthermore, in the case of pregnant women, unlike other population groups, clinical practice guidelines recommend TBS regardless of symptomatology, i.e., in malaria endemic areas, prenatal screening should include TBS in all pregnant women, even in those asymptomatic ^8^.

**KEY MESSAGES**

Motivation for the study: there is a lack of research on the accuracy of diagnostic tests for gestational, placental, and congenital malaria. No research has been done to compile the available evidence on the diagnostic ability of the thick blood smear versus the polymerase chain reaction (PCR).

Main findings: sensitivity of thick blood smear was low (50 and 54%) and specificity high (99%). The combined accuracy rate (area under the curve) of the thick blood smear was excellent (95%) when compared to nested PCR and fair (78%) when compared to quantitative PCR.

Implications: grouping the evidence on the accuracy of thick blood smear versus molecular diagnostic methods for pregnancy-associated malaria allows identifying limitations for epidemiological surveillance programs based on the microscopic technique.

## MATERIALS AND METHODS

### Type of study

A systematic review of diagnostic tests was carried out following the PRISMA-DTA (Preferred Reporting Items for Systematic reviews and Meta-Analyses of Diagnostic Test Accuracy studies) reporting recommendations (Supplementary Material 1).

### Research question

Population: pregnant women, their placentas and newborns in malaria endemic areas.

Intervention or test evaluated: TBS consisting of blood smears stained with 5% or 10% Giemsa-Field with light microscopy reading.

Comparator or standard test: nested or quantitative PCR, which is the most widely used molecular diagnostic method, with detection limit <0.02 parasites/µL.

Outcome: number of true positives and negatives, and number of false positives and negatives of TBS compared to PCR, in order to estimate the following diagnostic evaluation parameters: sensitivity, specificity, positive (PLR) and negative (NLR) likelihood ratio, odds ratio for diagnosis (ORD) and area under the ROC curve.

### Search and selection of studies

The search was performed in the multidisciplinary databases PubMed, SciELO, ScienceDirect, CINAHL (OVID EMCare) and Campbell-Cochrane Library; specialized databases for evaluation of diagnostic tests such as ARIF, HTA and DARE, and was complemented with a search in Google Scholar. For the selection of terms, we used the descriptors in health sciences (DeCS), Medical Subject Heading (MeSH) and a pearl harvesting (Pearl Growing) in reviews on PAM, obtaining four groups of terms: a) for infection: Malaria, *Plasmodium*, and “paludismo”; b) for study group: gestation, pregnancy, placental, and congenital; c) for diagnostic tests: PCR, TBS, microscopy, and microscopic; and d) for diagnostic parameters: accuracy, utility, sensitivity, and specificity. The combination of the terms resulted in six search strategies applied in Spanish and English in the nine databases, for a total of 108 syntaxes (Supplementary Material 2). In addition, we manually reviewed the bibliographic references of the selected studies to include those that met the eligibility criteria. The articles obtained from the searches (restricted to contain the terms in title or abstract), were saved in a common file in Zotero, to eliminate duplicates.

### Screening and eligibility

We screened studies published from 2010 to 2022 (the last update of the search and selection protocol was carried out on April 20, 2022), including original research (thus eliminating review studies, editorials, or book chapters), conducted on pregnant women, placentas or newborns, and whose objective was to report parameters of diagnostic accuracy of TBS (eliminating studies of test standardization, analytical validity or where the standard was not PCR). These criteria were applied independently by two investigators. In the eligibility phase, we excluded studies that did not use molecular diagnosis with PCR and those that only evaluated rapid tests that used histopathology as the reference standard.

### Data extraction

The following variables were extracted from the selected studies: title, author, year of publication, place of study, description of the population, sample size, description of the evaluated test and of the reference standard, number of true positives, true negatives, false positives, and false negatives.

### Methodological quality assessment and reproducibility analysis

The methodological quality assessment was carried out using the QUADAS (Quality Assessment of Diagnostic Accuracy Studies) guide. The reproducibility analysis was conducted independently by two researchers in order to guarantee concordance in all stages of the search and selection of studies, as well as in the extraction of information. Differences were resolved by consensus and subsequent review by a third investigator.

### Statistical analysis

Analyses were carried out in MetaDisc Software with estimation of the following diagnostic evaluation parameters: sensitivity, specificity, PLR, NLR, ORD, and area under the ROC curve, with their 95% confidence intervals (95% CI). Heterogeneity was determined with the Der Simonian-Laird Q statistic (χ^2^ distribution) (we chose a random-effects model in the presence of heterogeneous data) and uncertainty (sensitivity analysis) with the percentage weight of each study on the overall result. Meta-regressions were carried out to compare these diagnostic assessment parameters between asymptomatic women and those enrolled during prenatal care. We did not compare the diagnostic accuracy of TBS between gestational, placental, and congenital malaria since for the latter two, only one or two studies reported diagnostic parameters.

## RESULTS

**Figure 1:**
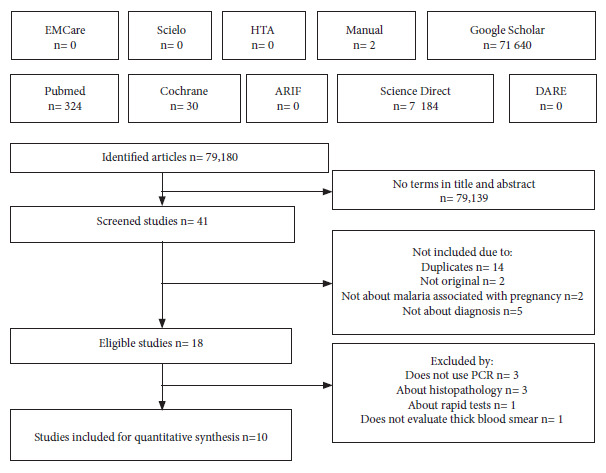
Flowchart of the search and selection of articles.

The search terms, without using filters in the databases, generated 79,180 results and only 41 containing these terms in the title and/or abstract were screened, of which ten fulfilled the search and selection protocol (Figure 1) (Supplementary Material 3).

The studies were conducted in seven African countries, one in the Americas and one in Asia, in 5691 pregnant women, 1415 placentas and 84 neonates. Most used nested polymerase chain reaction (nPCR) as a reference test, mainly with women captured during prenatal care (Table 1).

**Table 1:** Description of the studies included in the systematic review.

Author	Year^ a^	Country	Population
Standard: nested PCR			
Campos I ^17^	2011 (2004-2007)	Colombia	84 at delivery. 84 placentas and 84 neonatal samples. Mean age 23.1±5.2 years, independent of symptoms and gestational age. Enrolled in prenatal care or at delivery. Prospective parallel study. Sample size calculation for an expected prevalence of gestational malaria of 10%.
Minja D^ (^ ^18^	2012 (2008-2010)	Tanzania	924 with gestational age ≤24 weeks. 650 placentas (only 442 placental blood samples that had a PCR result could be evaluated). Women with 1-2 pregnancies with mean age 22.6±4.2, and multigravid with mean age 30.7 ± 5.3. Enrolled in villages, regardless of symptomatology. Prospective study of a cohort of pregnant women until delivery.
Kashif A ^19^	2013 (2012)	Sudan	156 febrile women (150 placentas not included in the diagnostic evaluation), mean age 27 years, average gestational age 19 weeks. They were enrolled during prenatal care. Two cross-sectional studies, one with pregnant women and the other with placentas.
Matangila J ^20^	2014 (2012)	Congo	332 patients enrolled during prenatal care (PCR was performed in 50%), asymptomatic and with anemia. Median age 27 years, mean gestational age 22 weeks, primigravida and multigravida. Prevalence study and cross-sectional survey.
Ahmed R ^21^	2015 (2012)	Indonesia	934 women aged ≥15 years, regardless of pregnancy and symptomatology. Cross-sectional study in 45 villages representing about 30000 exposed persons.
Kyabayinze D^ (^ ^22^	2016 (2010-2012)	Burkina Faso and Uganda	990 enrolled in prenatal care, second and third trimester of pregnancy, ≥16 years (with subsequent visits increased to 1742) independent of the presence of symptoms. Prospective multicenter cohort study, with sample size calculation for predictive values.
Vásquez A^ (^ ^23^	2018 (2016-2017)	Colombia	275 enrolled in prenatal care or delivery, symptomatic (mainly anemia), ≥ 15 years, any gestational age. 256 maternal peripheral blood samples at delivery and 256 placentas. Prospective descriptive study, part of a larger cross-sectional project.
Standard: quantitative PCR			
Mayor A ^24^	2012 (2003-2005)	Mozambique	272 at delivery and 272 placentas with an average age of 23 years and three previous pregnancies, regardless of the presence of symptoms. Retrospective study with data from a controlled trial (vs. placebo) on intermittent preventive treatment.
Umbers A ^25^	2015 (2010-2013)	Papua New Guinea	876 samples collected during prenatal care <26 weeks of gestation, ≥ 16 years, asymptomatic, (1162 samples for follow-up of some pregnant women). 158 placentas (only analyzed by histology). Prospective cohort study comparing various intermittent preventive treatment schemes.
Vásquez A ^26^	2020 (2017-2018)	Colombia	858 women aged ≥15 years, asymptomatic and with a mean gestational age of 22 weeks, received prenatal care. Cross-sectional study in two endemic municipalities, with sample size calculation for a sensitivity and specificity of 90% and prevalence of gestational malaria of 5%.

a Year of publication (year of execution).

Regarding the evaluation of the methodological quality of the included studies, all of them met between 90 or 100% of the 14 criteria of the QUADAS guidelines, showing excellent methodological quality. The only criterion that was not applied in all the studies was related to the selection of a complete population or a random sample, an aspect that was not clear in only one study ^18^.

We could not include the results of the study of Minja’s group ^18^ ^) ^in the meta-analysis since it presents the parameters of diagnostic evaluation in 442 samples without specifying the number of positive and negative for malaria. In this study microscopy showed a sensitivity of 70.8% (95%CI= 58.0-81.1) and specificity of 93.1 (95%CI=89.9-95.4). The diagnostic evaluation parameters (sensitivity, specificity, positive and negative likelihood ratios) for the remaining studies are presented in Table 2 as well as the percentage weight of each study in the combined measure obtained for studies comparing TBS against nPCR and quantitative polymerase chain reaction (qPCR).

**Table 2:** Description of the studies included in the systematic review.

Author	Sensitivity (IC95%)	Specificity (IC95%)	Positive likelihood ratio	Negative likelihood ratio	Weight percentage ^a^
Standard: nested PCR					
Campos ^17^	40.7 (22.4-61.2)	100 (93.7-100)	47.6 (2.9-779.8)	0.59 (0.44-0.81)	5-10
Kashif ^19^	94.4 (72.7-99.9)	100 (97.4-100)	256.1 (16.0-4086.1)	0.08 (0.02-0.37)	5-2
Matangila ^20^	67.3 (52.5-80.1)	97.4 (92.7-99.5)	26.3 (8.5-81.6)	0.33 (0.22-0.50)	12-9
Ahmed (Field) ^21^	32.3 (20.9-45.3)	98.4 (97.3-99.1)	20.1 (10.7-37.8)	0.69 (0.58-0.82)	14-12
Ahmed (Expert) ^21^	48.4 (35.5-61.4)	98.1 (96.9-98.9)	24.8 (14.5-42.4)	0.53 (0.41-0.67)	15-11
Kyabayinze (B.Faso - Antenatal) ^22^	39.8 (33.6-46.1)	99.8 (99.1-100)	166.6 (41.4-670.6)	0.60 (0.55-0.67)	11-13
Kyabayinze (B.Faso - Labor) ^22^	34.5 (28.6-40.8)	100 (99.6-100)	580.6 (36.2-9323.3)	0.65 (0.60-0.72)	5-13
Kyabayinze (Uganda - Antenatal) ^22^	69.7 (63.2-75.7)	98.4 (96.7-99.4)	43.5 (20.8-91.2)	0.31 (0.25-0.38)	14-12
Kyabayinze (Uganda - Labor) ^22^	54.1 (47.3-60.9)	99.5 (98.4-99.9)	118.3 (29.5-473.9)	0.46 (0.40-0.53)	11-12
Vásquez ^23^	79.5 (63.5-90.7)	100 (99.3-100)	776.5 (48.4-12454)	0.21 (0.12-0.39)	5-6
Global measurement	50.1 (47.2-53.0)	99.1 (98.8-99.4)	64.2 (29.2-140.9)	0.47 (0.38-0.58)	
Heterogeneity Chi^2^ (p)	117 (<0.001)	51 (<0.001)	39 (<0.001)	98 (<0.001)	
Estándar: PCR cuantitativa					
Mayor^ (^ ^24^	35.7 (26.3-46.0)	100 (97.9-100)	125.5 (7.8-2023.8)	0.64 (0.56-0.75)	28-36
Umbers^ (^ ^25^	63.7 (56.1-70.9)	97.6 (96.4-98.4)	26.3 (17.5-39.7)	0.37 (0.31-0.45)	44-34
Vásquez^ (^ ^26^	59.0 (42.1-74.4)	100 (99.6-100)	963.5 (59.6-15579)	0.41 (0.28-0.60)	28-30
Global measurement	54.2 (48.5-59.9)	98.8 (98.2-99.2)	112 (9.9-1267,9)	0.47 (0.29-0.74)	
Heterogeneity chi^2^ (p)	21 (<0.001)	34 (<0.001)	9 (0.013)	27 (<0.001)	

a The first value corresponds to the positive likelihood ratio and the second to the negative likelihood ratio. 95%CI: 95% confidence interval, PCR: polymerase chain reaction.

**Figure 2:**
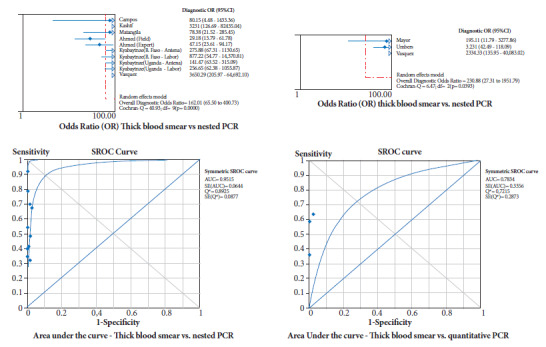
Diagnostic odds ratio and area under the ROC curve for thick blood smear comparison with nPCR (nested) and qPCR (quantitative).

Both in the subgroup that used nPCR as the standard and in the studies that used qPCR, the diagnostic accuracy results were statistically similar (confidence interval limits share values), with very low sensitivity (50 and 54% respectively), excellent specificity (99% in both cases), excellent PLR and poor NLR, with the evidence that no study had a statistically higher weight than the others in the overall or combined measure (Table 3). Using nPCR, the diagnostic OR was 162 (95%CI=66-401) and the area under the ROC curve was 0.9515, while with qPCR the diagnostic OR was 231 (95%CI=27-1951) and the area under the ROC curve was 0.7834 (Figure 2).

**Table 3:** Meta-analysis of the diagnostic accuracy of thick blood smear compared to PCR for gestational malaria in some subgroups of interest.

Diagnostic accuracy parameter	TBS Vs nPCR		TBS Vs qPCR
	Asymptomatic ^a^	Asymptomatic prenatal control ^b^	Asymptomatic prenatal control ^c^
Sensibility	49.4 (46.5-52.3)	52.2 (48.2-56.1)	62.9 (55.9-69.4)
Specificity	99.1 (98.8-99.3)	98.6 (98.2-99.0)	98.7 (98.0-99.1)
Positive LR	59.0 (26.5-132)	34.6 (17.6-67.9)	123 (3.4-4521)
Negative LR	0.48 (0.40-0.59)	0.48 (0.35-0.65)	0.38 (0.32-0.45)
Diagnostic OR	135 (55-331)	77 (33-183)	306 (10-9447)

a Only the study by Kashif ^19^, which evaluated febrile women, is excluded; it does not discriminate between those enrolled furing prenatal control and at delivery.

b The study by Vásquez ^23^ is excluded because the results do not discriminate between the results found in the women who were enrolled during prenatal control and at the time of delivery. In the studies by Matangila ^20^, Ahmed ^21^ and Kyabayinze ^22^, only the prenatal care group is included.

c Only the studies by Umbers ^25^ and Vasquez ^26^ are included.

TBS: thick blood smear, nPCR: nested polymerase chain reaction, qPCR: quantitative polymerase chain reaction, Positive LR: positive likelihood ratio, Negative LR: negative likelihood ratio, diagnostic OR: diagnostic odds ratio.

When the diagnostic evaluation parameters were disaggregated among more homogeneous and comparable groups (asymptomatic women under prenatal control), better values were obtained for all TBS parameters compared to qPCR, but these differences were not statistically significant. Overall, the sensitivity of TBS and NLR was low in both nPCR and qPCR comparisons, while specificity, PLR and diagnostic OR showed excellent results, but at the expense of high specificity (Table 3).

Finally, the study by Vasquez ^23^, by using nPCR, reported sensitivity and specificity of 100% (95%CI: 85.7-100.0) in the subgroup of symptomatic women, and sensitivity of 50% (95%CI: 28.0-72.0) and specificity of 100% (95%CI: 99.1-100. 0) in asymptomatic women; similar to Kashif ^19^ who reported sensitivity values of 94.4% (95%CI: 75.5-99.7) and specificity of 100% (95%CI: 98.0-100) in febrile women. Regarding congenital malaria, the article by Campos ^17^ was the only one that took umbilical cord blood and when compared with nPCR; they found sensitivity and specificity results of 0 and 97% for TBS. Similarly, few studies reported results of TBS accuracy in placental malaria; Kyabayinze ^22^ presented disaggregated findings for Burkina Faso and Uganda, obtaining sensitivity of 34.6% (95%CI: 20.4-58.7) and 54.0% (95%CI: 44.5-65.6), and specificity of 100% (95%CI=99.3-100) and 99.5% (95CI%: 96.6-99.9, respectively. The study by Mayor *et al*. ^24^ showed a sensitivity of 37.7% (95%CI: 28.1-47.3) for the Giemsa-stained placental smear and 35.7% (95%CI: 26.2-45.2) for the TBS, with a specificity of 99%.

## DISCUSSION

This systematic review evaluated the diagnostic accuracy of TBS in comparison with the PCR molecular test for PAM; we enrolled 5691 pregnant women, 1415 placentas and 84 neonates from Africa, Asia, and America. All Latin American studies took place in Colombia. Our study, in accordance with evidence-based medicine, conducted a rigorous search and an exhaustive analysis of the accuracy and safety provided by microscopy for the correct diagnosis of PAM. By covering as many databases and studies as possible, we found important deficiencies for the detection of infected pregnant women, if the prenatal control is based only on the application of TBS.

A previous meta-analysis compared PCR as an index test versus microscopy as reference and showed a sensitivity of 98% and a specificity of 65%, but it is not clear whether the low specificity was due to cases that were not detected by microscopy or whether they were false-positive PCR results ^15^. This study shows that when comparing TBS as an index test versus nPCR and qPCR as reference tests, the overall sensitivity was 50.1 (95%CI: 47.2-53.0) and 54.2 (95%CI: 48.5-59.9) respectively, with overall NLR of 0.47 in both cases. All the included studies show similar results, with no differences in the combined measures in the different subgroups (by type of PCR, prenatal control, or delivery, etc.), with low sensitivity and NLR, but high specificity, PLR, and ORD (the latter at the expense of high specificity). Taken together, it could be stated that the results regarding these parameters show difficulties for the application of this test in PAM, due to its disadvantages in capturing the real patients ^27^. In addition, there are other disadvantages of TBS compared to molecular techniques, such as its dependence on the expertise of the observer, which is demonstrated in the Umbers study by obtaining better results in the observation of professionals with experience in this field ^25^.

The described aspects of TBS are of concern, since there is evidence of a high proportion of false negatives for PAM, and a low diagnostic accuracy, mainly in asymptomatic pregnant women. It should be noted that the infected population generally has low and asymptomatic parasitemia in PAM, which would make them practically undetectable for conventionally used tests based on light microscopy. Low parasitemia has been attributed to the role of the placenta, where physiological processes such as the capture of infected erythrocytes by binding to chondroitin sulfate A, the adaptation of the immune system during gestation, the effect of previous pregnancies on the immune response to Plasmodium, among other morphological and physiological processes add to (and occasionally explain) the problem of TBS accuracy in this population affected by PAM ^28^.

Meta-analytic results of diagnostic evaluation for placental and congenital malaria are not presented because of the insufficient number of studies to estimate a combined measure. Despite this, the few available data show great deficiencies of TBS for these two clinical forms of malaria, even in the congenital form, sensitivity is found to be 0% ^17^, which agrees with a meta-analysis of Colombian studies in which the frequency of congenital malaria with TBS was 1% and 16% for PCR ^10^. In the case of placental malaria, it is important to highlight that the ability to detect cases with TBS was very low compared to PCR, which is of concern given that in this clinical presentation the diagnostic standard is histopathology; in this sense, further studies should evaluate the diagnostic accuracy of both tests (TBS and PCR) against the correct standard.

The shortcomings of TBS for the diagnosis of gestational malaria in endemic areas are even more serious when considering that this disease occurs in low-income areas where it is difficult to implement molecular tests (due to their higher cost compared to TBS). As a consequence, there is a high underreporting of cases with TBS and a high number of untreated infected pregnant women who may progress to present deleterious effects in the fetus, neonate, and mother, such as anemia, cerebral malaria, severe malaria, death, among others ^1^ ^,^ ^2^. All this is even more relevant when considering that, among the WHO objectives for PAM is the control and eradication of this disease, which will not be achieved through screening and diagnosis with TBS. In addition to these difficulties regarding diagnostic accuracy, there is a shortage of resources to achieve effective prevention of infection in pregnant women, through classic strategies such as the use of mosquito nets, mitigation of risk conditions in homes ^29^, access to prophylactic drugs in the areas, among others ^30^ ^,^ ^31^.

Despite the completeness of the protocol used for this systematic review, we found very few studies on diagnostic evaluation of TBS, probably because it is the standard test recommended for diagnosis in most international guidelines. This findings could also be supported by the fact that, in this field of diagnostic evaluation for malaria, studies that analyze rapid diagnostic tests are predominant, which are gaining strength by focusing on specific parasite molecules and allowing differentiation of *Plasmodium* species. In this regard, it is important to note that the technical recommendations for the diagnosis and follow-up of malaria treatment in endemic countries such as Colombia ^32^ suggest the use of TBS and rapid tests, despite their low sensitivity in asymptomatic patients with low parasitemia, given the difficulties in implementing molecular diagnosis in areas far from population centers. In these contexts, the use of molecular tests is restricted to symptomatic cases with negative TBS and rapid tests, in the face of discordant results and in infections by species other than *P. falciparum* or *P. vivax*, to support quality control of the diagnostic program and to monitor areas of low transmission. Based on the results of this meta-analysis, it is important to expand the use of molecular tests for the surveillance of PAM where the frequency of asymptomatic and submicroscopic infections is high ^10^.

Most of the meta-analyzed articles used nPCR as the standard and very few qPCR, this could be attributed to the high cost and greater complexity in the infrastructure required and in the protocols for this type of test. Blanquicet *et al*. ^33^ report that, although both diagnostic tests lead to confirmatory results, qPCR continues to be a test that provides more confidence and security in the timely diagnosis of PAM, since by quantifying the parasite, it allows the detection and follow-up of cases with a parasitemia lower than that detected by microscopy (10-30 parasites/µL). Another advantage of qPCR is the fact of having less risk of contamination, due to the fact that nPCR requires at least two steps and is an open system, contrary to qPCR which is developed in a closed thermal cycler ^33^ ^,^ ^34^.

In addition, qPCR is called a subvariant of real-time PCR, which in turn is an improvement of nPCR. It is possible to detect genus and species in these three types of PCR, but in the first two the quantification by means of Ct or cycle threshold reaches very low parasitemia values as demonstrated in the study of Khainar *et al*. ^35^ who found a greater analytical sensitivity of qPCR in four species of *Plasmodium*, with detection limits of 11 copies of rDNA/µl.

One of the main limitations of this study is the impossibility to deepen in some meta-regressions that would allow the role of variables such as the presence of symptoms (anemia or fever), the number of pregnancies, the collection of samples at delivery, among other clinical and gynecological characteristics, because the few studies that allude to these characteristics did not segregate the accuracy parameters according to these variables, or the number of those that did do so was very low, preventing the estimation of combined measures. For these same reasons it was not possible to meta-analyze the diagnostic accuracy of TBS for placental and congenital malaria. These limitations constitute the main lines of work for further studies in this field.

In conclusion, by means of an exhaustive protocol, we evidenced the low development of research on the diagnostic accuracy of TBS in PAM. Despite this, we managed to meta-analyze the few studies available, achieving a high number of evaluated pregnant women in whom it was demonstrated that microscopy has a poor performance for the diagnosis of asymptomatic or low parasitemia infections, which reinforces the importance of implementing other types of techniques in the monitoring and control of malaria infections in pregnant women, in order to achieve control and possible elimination of PAM.
