# Supplementary material for: Systematic review of the diagnostic accuracy of thick smear compared to polymerase chain reaction for pregnancy-associated malaria, 2010-2022
Source: Rev Peru Med Exp Salud Publica. 2022 Sep 30;39(3):302–11. doi: 10.17843/rpmesp.2022.393.11739 (PMC11397769; doi:10.17843/rpmesp.2022.393.11739)
Supplement: Supplementary files. — Supplementary material. [file rpmesp-39-03-11739-s001.zip › AO11739_MS/Material suplementario 2.docx]

**Revisión sistemática de la validez diagnóstica de la gota gruesa en comparación con PCR, para malaria asociada al embarazo, 2010-2022**

**Material suplementario 2. Sintaxis de búsqueda aplicadas en las bases de datos consultadas.**

| **PubMed** |
| --- |
| 1. ((((Malaria[Title/Abstract] OR Plasmodium[Title/Abstract] OR Paludism[Title/Abstract])) AND ((pregnancy[Title/Abstract] OR gestation[Title/Abstract]))) AND ((PCR[Title/Abstract] AND (TBS[Title/Abstract] OR microscopic[Title/Abstract] OR microscopy[Title/Abstract])))) AND ((validity[Title/Abstract] OR utility[Title/Abstract])) |
| 1. (((Malaria[Title/Abstract] OR Plasmodium[Title/Abstract] OR Paludism[Title/Abstract]) AND (pregnancy[Title/Abstract] OR gestation[Title/Abstract])) AND (PCR[Title/Abstract] AND (TBS[Title/Abstract] OR microscopic[Title/Abstract] OR microscopy[Title/Abstract]))) AND (sensitivity[Title/Abstract] OR specificity[Title/Abstract]) |
| 1. (((Malaria[Title/Abstract] OR Plasmodium[Title/Abstract] OR Paludism[Title/Abstract]) AND (placenta[Title/Abstract])) AND (PCR[Title/Abstract] AND (TBS[Title/Abstract] OR microscopic[Title/Abstract] OR microscopy[Title/Abstract]))) AND (validity[Title/Abstract] OR utility[Title/Abstract]) |
| 1. (((Malaria[Title/Abstract] OR Plasmodium[Title/Abstract] OR Paludism[Title/Abstract]) AND (placenta[Title/Abstract])) AND (PCR[Title/Abstract] AND (TBS[Title/Abstract] OR microscopic[Title/Abstract] OR microscopy[Title/Abstract]))) AND (sensitivity[Title/Abstract] OR specificity[Title/Abstract]) |
| 1. (((Malaria[Title/Abstract] OR Plasmodium[Title/Abstract] OR Paludism[Title/Abstract]) AND (congenita[Title/Abstract])) AND (PCR[Title/Abstract] AND (TBS[Title/Abstract] OR microscopic[Title/Abstract] OR microscopy[Title/Abstract]))) AND (validity[Title/Abstract] OR utility[Title/Abstract]) |
| 1. (((Malaria[Title/Abstract] OR Plasmodium[Title/Abstract] OR Paludism[Title/Abstract]) AND (congenita[Title/Abstract])) AND (PCR[Title/Abstract] AND (TBS[Title/Abstract] OR microscopic[Title/Abstract] OR microscopy[Title/Abstract]))) AND (sensitivity[Title/Abstract] OR specificity[Title/Abstract]) |
| **Science Direct** |
| Title, abstract, keywords: (Malaria OR Plasmodium OR Paludism) AND (pregnancy OR gestation) AND ((PCR AND (TBS OR microscopic OR microscopy)) AND (validity OR utility) ** |
| **Campbell Collaboration /Cochrane Library** |
| (Malaria OR Plasmodium OR Paludism) in Title Abstract Keyword AND (pregnancy OR gestation) in Title Abstract Keyword AND (PCR AND (TBS OR microscopic OR microscopy)) in Title Abstract Keyword AND (validity OR utility) in Title Abstract Keyword-(Word variations have been searched) ** |
| **Scielo, EMCare ARIF, HTA y DARE** |
| (ab:( Malaria OR Plasmodium OR Paludism )) AND (ab:(pregnancy OR gestation )) AND (ab:(PCR AND (TBS OR microscopic OR microscopy) )) AND (ab:(validity OR utility))** |
| **Google Scholar** |
| allintitle: (Malaria OR Plasmodium OR Paludism) AND (pregnancy OR gestation) AND ((PCR AND (TBS OR microscopic OR microscopy) AND (validity OR utility) ** |

** Sólo se presenta la primera combinación de términos para explicitar las especificidades de las sintaxis de esta base de datos; cinco restantes corresponden a los demás términos descritos para PubMed. Estas búsquedas también se realizaron en español.
