# Supplementary material for: Systematic review of the diagnostic accuracy of thick smear compared to polymerase chain reaction for pregnancy-associated malaria, 2010-2022
Source: Rev Peru Med Exp Salud Publica. 2022 Sep 30;39(3):302–11. doi: 10.17843/rpmesp.2022.393.11739 (PMC11397769; doi:10.17843/rpmesp.2022.393.11739)
Supplement: Translation

# Translation: Revisión sistemática de la exactitud diagnóstica de la gota gruesa en comparación con la reacción en cadena de la polimerasa para malaria asociada al embarazo, 2010-2022

**Authors:** Roxana Gómez-Hoyos, Jaiberth Antonio Cardona-Arias, Luis Felipe Higuita Gutiérrez, Walter Salas-Zapata, Jaime Carmona-Fonseca

**Affiliations:** 1 Escuela de Microbiología, Universidad de Antioquia, Medellín, Colombia.; 2 Universidad de Antioquia, Medellín, Colombia.; 3 Universidad Cooperativa de Colombia, Medellín, Colombia.; 4 Grupo Salud y Comunidad César Uribe Piedrahíta, Facultad de Medicina, Universidad de Antioquia, Medellín, Colombia.

**Keywords:** Gota Gruesa, PCR, Exactitud Diagnóstica, Malaria Asociada al Embarazo, Metaanálisis

## Abstract

**Objetivos.:**

Evaluar la exactitud de gota gruesa (GG) frente a la reacción en cadena de la polimerasa (PCR) cuantitativa para la malaria asociada al embarazo (MAE).

**Materiales y métodos.:**

Se realizó una revisión sistemática de pruebas diagnósticas en nueve bases de datos. Se evaluó la calidad metodológica con QUADAS. Se estimó sensibilidad, especificidad, cociente de probabilidad positivo (CPP) y negativo (CPN), razón de *odds* diagnóstica (ORD) y área bajo la curva ROC. Se determinó la heterogeneidad con el estadístico Q de Der Simonian-Laird y la incertidumbre con el porcentaje de peso de cada estudio sobre el resultado global.

**Resultados.:**

Se incluyeron diez estudios con 5691 gestantes, 1415 placentas y 84 neonatos. En los estudios con nPCR (PCR anidada) y qPCR (PCR cuantitativa) como estándar, los resultados de exactitud diagnóstica fueron estadísticamente similares, con sensibilidad muy baja (50 y 54%, respectivamente), alta especificidad (99% en ambos casos), alto CPP y deficiente CPN. Usando nPCR la OR diagnóstica fue 162 (IC95%=66-401) y el área bajo la curva ROC fue 95%, mientras que con qPCR fueron 231 (IC95%=27-1951) y 78%, respectivamente.

**Conclusiones.:**

Mediante un protocolo exhaustivo se demostró el bajo desarrollo de investigaciones sobre la exactitud diagnóstica de la GG en MAE. Se demostró que la microscopía tiene un desempeño deficiente para el diagnóstico de infecciones asintomáticas o de baja parasitemia, lo que afianza la importancia de implementar otro tipo de técnicas en el seguimiento y control de las infecciones por malaria en las gestantes, con el fin de lograr el control y posible eliminación de la MAE.

## INTRODUCCIÓN

La infección por *Plasmodium* spp. durante el embarazo (malaria asociada al embarazo o MAE) incluye tres cuadros clínicos: malaria gestacional (MG) o infección demostrada en la sangre periférica de la madre; malaria placentaria (MP) o presencia de *Plasmodium *spp. en este órgano, y malaria congénita (MC) que corresponde a la infección del recién nacido durante la vida intrauterina o el parto, con presencia de *Plasmodium *spp. en los primeros siete días de vida o más tardíamente, con manifestaciones clínicas entre los 10-30 días posparto ^1^ ^-^ ^5^. La MAE tiene graves riesgos para la gestante como anemia, malaria cerebral, malaria grave y muerte; además, en el feto y en el recién nacido ocasiona desenlaces negativos como retraso del crecimiento intrauterino, anemia, muerte intrauterina, parto prematuro, bajo peso al nacer, entre otros ^1^ ^,^ ^2^.

La Organización Mundial de la Salud (OMS) indica que África subsahariana presenta una prevalencia moderada-alta de MAE, estimándose que de 33,2 millones de embarazos el 35% estuvo expuesto a malaria; asimismo, en África Central existe un 40% de riesgo de transmisión de malaria en las gestantes ^6^. En América, el 1% de los casos de malaria reportados entre 2010 y 2016 se presentó en embarazadas, causando catorce defunciones en este grupo poblacional ^7^.

La gota gruesa (GG) es la prueba diagnóstica más utilizada para la malaria y es la prueba estándar o patrón en las zonas de endemia. Esto se puede corroborar en algunas guías de práctica clínica de la Organización Panamericana de la Salud y de la Sociedad Española de Medicina Tropical y Salud Internacional (SEMTSI) ^8^ ^,^ ^9^. No obstante, esta prueba genera una alta subestimación de casos frente a lo hallado con pruebas moleculares. Por ejemplo, un metaanálisis en Colombia concluyó que al usar la GG para detección de MAE la prevalencia fue 4,5% (IC95%: 2,9-6,9), con MG de 5,8% (IC95%: 3,8-8,7), MP 3,4% (IC95%: 1,7-6,7) y MC 1,3% (IC95%: 0,6-3,0); mientras que al usar PCR la prevalencia de MAE asciende a 14,4% (IC95%=7,6-25,5), MG 16,7% (IC95%: 9,0-28,8), MP 11,0% (IC95%: 4,1-26,3) y MC 16,2% (IC95%: 8,2-29,5) ^10^. Sin embargo, un estudio en Colombia sobre malaria en general realizado con 314 muestras presentó resultados diferentes al reportar una sensibilidad del 97% y una especificidad del 100% comparando GG realizadas en los puestos de microscopía con PCR del laboratorio de referencia. Es importante mencionar que esta investigación se realizó en población sintomática que no refleja las condiciones de la población general y no capta todo el espectro clínico de la enfermedad desde los estadios asintomáticos ^11^.

En adición a lo anterior, se debe tener presente que la GG presenta problemas de sensibilidad en infecciones de baja densidad parasitaria y asintomáticas, mientras que esta situación no se presenta o es mucho menor con el uso de métodos moleculares como la PCR. Incluso, se ha documentado la importancia de la PCR para detectar y tratar individuos con parasitemias bajas que pueden actuar como reservorios de transmisión y obstaculizar las metas de eliminación de la enfermedad ^12^. En general, la GG se considera el estándar de referencia para malaria; sin embargo, para el caso de MAE es más pertinente tomar como estándar la PCR por las siguientes razones: a) en MAE se presenta una elevada frecuencia de infecciones submicroscópicas, de baja densidad y asintomáticas, que no se captan con GG, b) la GG tiene un límite de detección más deficiente que la PCR (10-30 parásitos/microlitro de sangre, pero en condiciones de campo esta cifra puede ascender a 50-100 parásitos/microlitro de sangre), c) la GG es adecuada para la malaria clínica (sintomática), pero no para la asintomática, por lo que no es adecuado tomarla como estándar para los programas de vigilancia epidemiológica), y d) los avances de la última década en el diagnóstico molecular evidencia la mejor capacidad diagnóstica de la PCR ^10^ ^,^ ^11^ ^,^ ^13^ ^,^ ^14^.

Pese a las discrepancias en la detección de *Plasmodium* spp. con GG y PCR, en la literatura científica no se dispone de una investigación que haya sintetizado y evaluado la evidencia disponible sobre la exactitud diagnóstica de GG en la malaria gestacional, placentaria o congénita; incluso, para otros grupos poblaciones son escasos los estudios sobre exactitud y desempeño diagnóstico de esta prueba. Además, no se encuentran revisiones sistemáticas relacionadas con el tema; se dispone de un estudio que sistematizó las investigaciones sobre precisión diagnóstica de pruebas para malaria asociada al embarazo; sin embargo, esta se focalizó en pruebas rápidas e incluyó estudios entre 1914 y 2009 ^15^, pero los principales avances de diagnóstico molecular son posteriores al 2009.

Por lo anterior, se realizó una revisión sistemática con el objetivo de evaluar la exactitud diagnóstica de la GG frente a la PCR para la MAE, 2010-2022. Este estudio es relevante para mejorar prácticas clínicas, generar evidencia de mejor calidad, estimar parámetros de exactitud diagnósticas con mayor precisión y posibilidad de extrapolación, consolidar hipótesis sobre la necesidad de mejorar métodos diagnósticos, incrementar la exactitud de las conclusiones de estudios individuales e identificar áreas de incertidumbre donde sea necesario realizar investigación adicional ^16^. Además, en el caso de las gestantes, a diferencia de los demás grupos poblacionales, las guías de práctica clínica recomiendan la realización de la GG independientemente de la sintomatología, es decir, en las zonas endémicas de malaria, el control prenatal debe incluir la GG en todas las gestantes, incluso en las asintomáticas ^8^.

**MENSAJE CLAVE**

Motivación para realizar el estudio: existen pocas investigaciones sobre la exactitud de las pruebas diagnósticas para malaria gestacional, placentaria y congénita. No se ha realizado una investigación que agrupe la evidencia disponible sobre la capacidad diagnóstica de la gota gruesa frente a la reacción en cadena de la polimerasa (PCR).

Principales hallazgos: la sensibilidad de la gota gruesa fue baja (50 y 54%) y la especificidad alta (99%). El índice combinado de exactitud (área bajo la curva) de la gota gruesa fue excelente (95%) cuando se comparó con la PCR anidada y regular (78%) en la comparación con la PCR cuantitativa.

Implicancias en salud pública: agrupar la evidencia sobre la exactitud de gota gruesa frente a métodos de diagnóstico molecular para malaria asociada con el embarazo permite identificar limitaciones para los programas de vigilancia epidemiológica basado en la técnica microscópica.

## MATERIALES Y MÉTODOS

### Tipo de estudio

Se realizó una revisión sistemática de pruebas diagnósticas siguiendo las recomendaciones para el reporte de PRISMA-DTA (Preferred Reporting Items for Systematic reviews and Meta-Analyses of Diagnostic Test Accuracy studies) (Material suplementario 1).

### Pregunta de investigación

Población: gestantes, sus placentas y recién nacidos de zonas endémicas de malaria.

Intervención o prueba evaluada: GG consistente en extendidos de sangre teñidas con Giemsa-Field al 5 o 10% con lectura en microscopio de luz.

Comparador o prueba estándar: PCR anidada o cuantitativa, las cuales constituyen el método de diagnóstico molecular más usado, con límite de detección <0,02 parásitos/µL.

Desenlace: número de verdaderos positivos y negativos, y número de falsos positivos y negativos de GG en comparación con PCR, para estimar los siguientes parámetros de evaluación diagnóstica: sensibilidad, especificidad, cociente de probabilidad positivo (CPP) y negativo (CPN), razón de *odds* diagnóstica (ORD) y área bajo la curva ROC.

### Búsqueda y selección de los estudios

La búsqueda se realizó en las bases de datos multidisciplinarias PubMed, SciELO, ScienceDirect, CINAHL (OVID EMCare) y Campbell-Cochrane Library; bases de datos especializadas para evaluación de pruebas diagnósticas como ARIF, HTA y DARE, y se complementó con una búsqueda en Google Scholar. Para la selección de los términos se usaron descriptores en ciencias de la salud (DeCS), Medical Subject Heading (MeSH) y una cosecha de perlas (Pearl Growing) en revisiones sobre MAE, obteniendo cuatro grupos de términos: a) para la infección: malaria, *Plasmodium* y paludismo; b) para el grupo de estudio: gestación, embarazo, placenta y congénita; c) para las pruebas diagnósticas: PCR, GG, microscopía y microscópico, y d) para los parámetros diagnósticos: exactitud, utilidad, sensibilidad y especificidad. La combinación de los términos dio lugar a seis estrategias de búsqueda aplicadas en español e inglés en las nueve bases de datos, para un total de 108 sintaxis (Material suplementario 2). Además, se hizo una revisión manual de las referencias bibliográficas de los estudios seleccionados, para incluir aquellos que cumplían los criterios de elegibilidad. Los artículos obtenidos con las búsquedas (restringidas a contener los términos en título o resumen), se guardaron en un archivo común en Zotero, para eliminar los duplicados.

### Tamización y elegibilidad

Se tamizaron estudios publicados desde el 2010 hasta el 2022 (con última actualización del protocolo de búsqueda y selección realizada el 20 de abril del 2022), investigaciones originales (con lo cual se eliminaron estudios de revisión, editoriales o capítulos de libros), realizadas en gestantes, placentas o recién nacidos, y cuyo objetivo fuese informar parámetros de exactitud diagnóstica de la GG (eliminando estudios de estandarización de pruebas, validez analítica o donde el estándar no fue PCR). Estos criterios fueron aplicados de manera independiente por dos investigadores. En la fase de elegibilidad, se excluyeron estudios que no utilizaron diagnóstico molecular con PCR, aquellas que solo evaluaron pruebas rápidas y donde el estándar de referencia fue la histopatología.

### Extracción de los datos

En los estudios seleccionados se realizó extracción de las siguientes variables: título, autor, año de publicación, lugar de estudio, descripción de la población, tamaño de la muestra, descripción de la prueba evaluada y del estándar de referencia, número de verdaderos positivos, verdaderos negativos, falsos positivos y falsos negativos.

### Evaluación de la calidad metodológica y análisis de reproducibilidad

La evaluación de la calidad metodológica se realizó con la guía QUADAS (Quality Assessment of Diagnostic Accuracy Studies). El análisis de reproducibilidad lo realizaron dos investigadores de forma independiente con el propósito de garantizar la concordancia en todas las etapas de la búsqueda y selección de los estudios, así como de la extracción de la información. Las diferencias se resolvieron por consenso y posterior revisión de un tercer investigador. 

### Análisis estadístico

Los análisis se realizaron en el *Software* MetaDisc con la estimación de los siguientes parámetros de evaluación diagnóstica: sensibilidad, especificidad, CPP, CPN, ORD y área bajo la curva ROC, con sus intervalos de confianza del 95% (IC95%). Se determinó la heterogeneidad con el estadístico Q (distribución χ^2^) de Der Simonian-Laird (ante datos heterogéneos se eligió un modelo de efectos aleatorios) y la incertidumbre (análisis de sensibilidad) con el porcentaje de peso de cada estudio sobre el resultado global. Se realizaron metarregresiones para comparar estos parámetros de evaluación diagnóstica entre mujeres asintomáticas y aquellas captadas durante el control prenatal. No se realizaron comparaciones de la exactitud diagnóstica de la GG entre malaria gestacional, placentaria y congénita dado que para estas dos últimas solo uno o dos estudios reportaron los parámetros diagnósticos.

## RESULTADOS

Los términos de búsqueda, sin utilizar filtros en las bases de datos generó 79 180 resultados y solo se tamizaron 41 que contenían estos términos en el título y/o resumen, de los cuales diez cumplieron el protocolo de búsqueda y selección (Figura 1) (Material Suplementario 3).

**Figura 1:** Flujograma de búsqueda y selección de los artículos.

Los estudios se realizaron en siete países africanos, uno de América y uno de Asia, en 5691 gestantes, 1415 placentas y 84 neonatos. La mayoría utilizó la reacción en cadena de la polimerasa anidada (nPCR) como prueba de referencia, principalmente con mujeres captadas durante la atención prenatal (Tabla 1).

**Tabla 1:** Descripción de los estudios incluidos en la revisión sistemática.

Autor	Año^ a^	País	Población
Estándar: PCR anidada			
Campos I ^17^	2011 (2004-2007)	Colombia	84 en el momento del parto. 84 placentas y 84 muestras de neonatos. Edad media 23,1±5,2 años, independiente de síntomas y edad gestacional. Captados en la atención prenatal o al momento del parto. Estudio prospectivo en paralelo. Cálculo de tamaño de muestra para una prevalencia esperada de malaria gestacional del 10%.
Minja D^ (^ ^18^	2012 (2008-2010)	Tanzania	924 con edad gestacional ≤24 semanas. 650 placentas (la evaluación sólo se pudo realizar en 442 muestras de sangre placentaria que tuvieron resultado de PCR). Mujeres con 1-2 embarazos con edad media 22,6±4,2, y multigrávidas con edad media 30,7 ± 5,3. Captados en aldeas, independiente de sintomatología. Estudio prospectivo de una cohorte de embarazadas hasta el parto.
Kashif A ^19^	2013 (2012)	Sudán	156 febriles (150 placentas no incluidas en la evaluación diagnóstica), edad media 27 años, y en promedio 19 semanas de gestación. Captadas en atención prenatal. Dos estudios transversales, uno con gestantes y otro con placentas.
Matangila J ^20^	2014 (2012)	El Congo	332 captadas en atención prenatal (la PCR se realizó al 50%) asintomáticas y con anemia. Mediana de edad de 27 años, edad gestacional promedio de 22 semanas, primigravidas y multigrávidas. Estudio de prevalencia y encuesta transversal.
Ahmed R ^21^	2015 (2012)	Indonesia	934 captadas en atención prenatal, ≥15 años, independiente de gravidez y sintomatología. Estudio transversal en 45 aldeas que representan cerca de 30000 personas expuestas.
Kyabayinze D^ (^ ^22^	2016 (2010-2012)	Burkina Faso y Uganda	990 captadas en atención prenatal, segundo y tercer trimestre del embarazo, ≥16 años (con visitas subsiguientes aumentaron a 1742) independiente de la presencia de síntomas. Estudio multicéntrico prospectivo de cohorte, con cálculo de tamaño de muestra para los valores predictivos.
Vásquez A^ (^ ^23^	2018 (2016-2017)	Colombia	275 captadas en atención prenatal o el parto, sintomáticas (principalmente anemia), ≥ 15 años, cualquier edad gestacional. 256 muestras de sangre periférica materna al momento del parto y 256 placentas. Estudio prospectivo descriptivo, parte de un proyecto mayor de tipo transversal.
Estándar: PCR cuantitativa			
Mayor A ^24^	2012 (2003-2005)	Mozambique	272 en el momento del parto y 272 placentas con un promedio de 23 años y de tres embarazos anteriores, independiente de la presencia de síntomas. Estudio retrospectivo con datos de un ensayo controlado (vs. placebo) sobre tratamiento intermitente preventivo.
Umbers A ^25^	2015 (2010-2013)	Papua Nueva Guinea	876 captadas en atención prenatal <26 semanas de gestación, ≥ 16 años, asintomáticas, (1162 muestras por seguimiento a algunas gestantes). 158 placentas (sólo analizadas por histología). Estudio prospectivo de cohorte que comparó varios esquemas de tratamiento intermitente preventivo.
Vásquez A ^26^	2020 (2017-2018)	Colombia	858 captadas en atención prenatal, ≥15 años, asintomáticas y con una media de edad gestacional de 22 semanas. Estudio transversal en dos municipios endémicos, con cálculo de tamaño de muestra para una sensibilidad y especificidad del 90% y prevalencia de malaria gestacional del 5%.

a Año de publicación (año de ejecución).

Respecto a la evaluación de la calidad metodológica de los estudios incluidos, todos cumplieron el 90 o 100% de los 14 criterios de la guía QUADAS, evidenciando una excelente calidad metodológica, el único criterio que no fue aplicado en la totalidad de estudios se relacionó con la selección de una población completa o una muestra aleatoria, aspecto que no fue claro en un único estudio ^18^.

En el metaanálisis no fue posible incluir los resultados del estudio del grupo de Minja ^18^ dado que presenta los parámetros de evaluación diagnóstica en 442 muestras sin especificar el número de positivos y negativos para malaria. En este estudio la microscopia presentó sensibilidad de 70,8% (IC95%= 58,0-81,1) y especificidad de 93,1 (IC95%=89,9-95,4). Los parámetros de evaluación diagnóstica (sensibilidad, especificidad, los cocientes de probabilidad positivos y negativos) para los estudios restantes se presentan en la Tabla 2 así como el porcentaje de peso de cada estudio en la medida combinada que se obtiene para los estudios que comparan GG contra nPCR y la reacción en cadena de la polimerasa cuantitativa (qPCR).

**Tabla 2:** Descripción de los estudios incluidos en la revisión sistemática.

Autor	Sensibilidad (IC95%)	Especificidad (IC95%)	Cociente de probabilidad positivo	Cociente de probabilidad negativo	Porcentaje de peso^ a^
Estándar: PCR anidada					
Campos ^17^	40,7 (22,4-61,2)	100 (93,7-100)	47,6 (2,9-779,8)	0,59 (0,44-0,81)	5-10
Kashif ^19^	94,4 (72,7-99,9)	100 (97,4-100)	256,1 (16,0-4086,1)	0,08 (0,02-0,37)	5-2
Matangila ^20^	67,3 (52,5-80,1)	97,4 (92,7-99,5)	26,3 (8,5-81,6)	0,33 (0,22-0,50)	12-9
Ahmed (Field) ^21^	32,3 (20,9-45,3)	98,4 (97,3-99,1)	20,1 (10,7-37,8)	0,69 (0,58-0,82)	14-12
Ahmed (Expert) ^21^	48,4 (35,5-61,4)	98,1 (96,9-98,9)	24,8 (14,5-42,4)	0,53 (0,41-0,67)	15-11
Kyabayinze (B.Faso - Antenatal) ^22^	39,8 (33,6-46,1)	99,8 (99,1-100)	166,6 (41,4-670,6)	0,60 (0,55-0,67)	11-13
Kyabayinze (B.Faso - Labor) ^22^	34,5 (28,6-40,8)	100 (99,6-100)	580,6 (36,2-9323,3)	0,65 (0,60-0,72)	5-13
Kyabayinze (Uganda - Antenatal) ^22^	69,7 (63,2-75,7)	98,4 (96,7-99,4)	43,5 (20,8-91,2)	0,31 (0,25-0,38)	14-12
Kyabayinze (Uganda - Labor) ^22^	54,1 (47,3-60,9)	99,5 (98,4-99,9)	118,3 (29,5-473,9)	0,46 (0,40-0,53)	11-12
Vásquez ^23^	79,5 (63,5-90,7)	100 (99,3-100)	776,5 (48,4-12454)	0,21 (0,12-0,39)	5-6
Medida global	50,1 (47,2-53,0)	99,1 (98,8-99,4)	64,2 (29,2-140,9)	0,47 (0,38-0,58)	
Heterogeneidad Chi^2^ (p)	117 (<0,001)	51 (<0,001)	39 (<0,001)	98 (<0,001)	
Estándar: PCR cuantitativa					
Mayor^ (^ ^24^	35,7 (26,3-46,0)	100 (97,9-100)	125,5 (7,8-2023,8)	0,64 (0,56-0,75)	28-36
Umbers^ (^ ^25^	63,7 (56,1-70,9)	97,6 (96,4-98,4)	26,3 (17,5-39,7)	0,37 (0,31-0,45)	44-34
Vásquez^ (^ ^26^	59,0 (42,1-74,4)	100 (99,6-100)	963,5 (59,6-15579)	0,41 (0,28-0,60)	28-30
Medida global	54,2 (48,5-59,9)	98,8 (98,2-99,2)	112 (9,9-1267,9)	0,47 (0,29-0,74)	
Heterogeneidad chi^2^ (p)	21 (<0,001)	34 (<0,001)	9 (0,013)	27 (<0,001)	

a El primer valor corresponde al cociente de probabilidad positivo y el segundo al cociente de probabilidad negativo.

IC95%: intervalo de confianza del 95%, PCR: reacción en cadena de la polimerasa.

Tanto en el subgrupo que empleó nPCR como estándar, como en los estudios que utilizaron qPCR, los resultados de exactitud diagnóstica fueron estadísticamente similares (los límites de los intervalos de confianza comparten valores), con sensibilidad muy baja (50 y 54% respectivamente), excelente especificidad (99% en ambos casos), excelente CPP y deficiente CPN, con la evidencia que ningún estudio tuvo un peso estadísticamente mayor que los demás en la medida global o combinada (Tabla 3). Usando nPCR la OR diagnóstica fue 162 (IC95%=66-401) y el área bajo la curva ROC fue 0,9515, mientras que con qPCR la OR diagnóstica fue 231 (IC95%=27-1951) y el área bajo la curva ROC fue 0,7834 (Figura 2).

**Tabla 3:** Metaanálisis de la exactitud diagnóstica de la gota gruesa en comparación con la PCR para malaria gestacional en algunos subgrupos de interés.

Parámetro de exactitud diagnóstica	GG Vs nPCR		GG Vs qPCR
	Asintomáticas ^a^	Control prenatal asintomáticas ^b^	Control prenatal asintomáticas ^c^
Sensibilidad	49,4 (46,5-52,3)	52,2 (48,2-56,1)	62,9 (55,9-69,4)
Especificidad	99,1 (98,8-99,3)	98,6 (98,2-99,0)	98,7 (98,0-99,1)
CP positivo	59,0 (26,5-132)	34,6 (17,6-67,9)	123 (3,4-4521)
CP negativo	0,48 (0,40-0,59)	0,48 (0,35-0,65)	0,38 (0,32-0,45)
OR diagnóstica	135 (55-331)	77 (33-183)	306 (10-9447)

a Solo se excluye el estudio de Kashif ^19^ que evaluó mujeres febriles, no se discrimina entre las captadas en control prenatal y el parto.

b Se excluye el estudio de Vásquez ^23^ ^) ^porque en los resultados no discrimina lo hallado en las mujeres captadas en control prenatal y al momento del parto. En los estudios de Matangila ^20^, Ahmed ^21^ y Kyabayinze ^22^, sólo se incluye el grupo de atención prenatal.

c Solo se incluyen los estudios de Umbers ^25^ y Vásquez ^26^.

GG: gota gruesa, nPCR: reacción en cadena de la polimerasa anidada, qPCR: reacción en cadena de la polimerasa cuantitativa, CP positivo: cociente de probabilidad positivo, CP negativo: cociente de probabilidad negativo, OR diagnóstica: odds ratio diagnóstica

**Figura 2:** *Odds ratio *diagnóstica y área bajo la curva ROC para la comparación de gota gruesa con nPCR (anidada) y qPCR (cuantitativa).

Al desagregar los parámetros de evaluación diagnóstica entre grupos más homogéneos y comparables (mujeres asintomáticas captadas en control prenatal), se obtuvieron mejores valores en todos los parámetros de la GG en comparación con qPCR, pero estas diferencias no son estadísticamente significativas. De manera general, tanto en las comparaciones con nPCR como qPCR la sensibilidad de la GG y el CPN es deficiente, mientras que la especificidad, el CPP y la OR diagnóstica presentan excelentes resultados, pero a expensas de la alta especificidad (Tabla 3).

Finalmente, el estudio de Vásquez ^23^, usando nPCR, reportó sensibilidad y especificidad del 100% (IC95%: 85,7-100.0) en el subgrupo de mujeres sintomáticas, y sensibilidad del 50% (IC95%: 28,0-72,0) y especificidad del 100% (IC95%: 99,1-100.0) en las asintomáticas; similar a Kashif ^19^ que reportó valores de sensibilidad de 94,4% (IC95%: 75,5-99,7) y de especificidad del 100% (IC95%: 98,0-100) en mujeres febriles. Respectó a la malaria congénita el artículo de Campos ^17^ fue el único que toma sangre de cordón umbilical y al realizar la comparación con nPCR se obtienen resultados de sensibilidad y especificidad de 0 y 97% para la GG. De manera similar, pocos estudios reportaron resultados de exactitud de GG en malaria placentaria; Kyabayinze ^22^ presentó hallazgos desagregado para Burkina Faso y Uganda, obteniendo sensibilidad de 34,6% (IC95%: 20,4-58,7) y 54,0% (IC95%: 44,5-65,6), y especificidad de 100% (IC95%=99,3-100) y 99,5% (IC95%: 96,6-99,9, respectivamente. Por su parte, el estudio de Mayor *et al.* ^24^ presenta una sensibilidad de 37,7% (IC95%: 28,1-47,3) en el frotis de placenta teñido con Giemsa y 35,7% (IC95%: 26,2-45,2) en la GG, con especificidad del 99%.

## DISCUSIÓN

En la presente revisión sistemática se evaluó la exactitud diagnóstica de la GG en comparación con la prueba molecular PCR para MAE; se reunió 5691 gestantes, 1415 placentas y 84 neonatos de África, Asia y América. De este continente, todos los estudios se realizaron en Colombia. El presente estudio, acorde con la medicina basada en la evidencia, realizó una búsqueda rigurosa y un análisis exhaustivo de la exactitud y seguridad que aporta la microscopia para el correcto diagnóstico de la MAE. Abarcando la mayor cantidad de bases de datos y estudios posibles, se evidenciaron importantes falencias para la captación de gestantes infectadas, si el control prenatal se basa solo en la aplicación de GG.

En un metaanálisis previo se comparó la PCR como prueba índice frente a la microscopía como prueba de referencia y los resultados muestran una sensibilidad de 98% y una especificidad de 65%, pero sin esclarecer si la baja especificidad fue producto de casos que no se detectaron en la microscopía o si fueron resultados de PCR falsos positivos ^15^. En este estudio se muestra que al comparar la GG como prueba índice frente la nPCR y qPCR como pruebas de referencia se halló una baja sensibilidad con una medida global de 50,1 (IC95%: 47,2-53,0) y 54,2 (IC95%: 48,5-59,9) respectivamente, con CPN globales de 0,47 en ambos casos. Todos los estudios incluidos muestran resultados similares, sin encontrar diferencias en las medidas combinadas en los diferentes subgrupos analizados (por tipo de PCR, captaciones en control prenatal o parto, etc.), con sensibilidad y CPN bajos, pero especificidad, CPP y ORD altos (estos últimos a expensas de la alta especificidad). De manera conjunta, se podría aseverar que los resultados obtenidos en estos parámetros muestran dificultades para la aplicación de esta prueba en MAE, debido a sus inconvenientes para captar los reales enfermos ^27^. A esto se sumarían otras desventajas de la GG frente a las técnicas moleculares, como su dependencia de la experticia del observador, lo cual es demostrado en el estudio de Umbers al obtener mejores resultados en la observación de profesionales con experiencia en este campo ^25^.

Los aspectos de la GG descritos previamente son preocupantes, dado que se evidencia una alta proporción de falsos negativos para MAE, y una baja exactitud diagnóstica, principalmente en gestantes asintomáticas. En este orden de ideas se debe recordar que en la MAE la población infectada generalmente presenta parasitemias bajas y asintomáticas, lo que las haría prácticamente indetectables para las pruebas convencionalmente usadas y basadas en microscopía óptica. Estas bajas parasitemias se han atribuido al rol de la placenta, donde posiblemente intervienen procesos fisiológicos como la captura de los eritrocitos infectados por unión al sulfato de condroitina A, la adaptación del sistema inmune durante la gestación, el efecto de embarazos previos sobre la respuesta inmune al *Plasmodium*, entre otros procesos morfológicos y fisiológicos que se suman (y ocasionalmente explicarían) al problema de exactitud de la GG en esta población afectada por MAE ^28^.

No se presentan resultados metaanalíticos de evaluación diagnóstica para malaria placentaria y congénita dado el número insuficiente de estudios para estimar una medida combinada. A pesar de esto, los pocos datos disponibles muestran grandes deficiencias de la GG para estas dos formas clínicas de la malaria, incluso en la forma congénita se halla sensibilidad de 0% ^17^, lo que concuerda con un metaanálisis de los estudios colombianos en el cual la frecuencia de malaria congénita con GG fue 1% mientras que al usar PCR fue 16% ^10^. En el caso de la malaria placentaria es importante resaltar que la capacidad de detectar casos con GG fue muy baja en comparación con PCR, lo que es preocupante dado que en esta presentación clínica el estándar diagnóstico es la histopatología; en tal sentido, estudios posteriores deberán evaluar la exactitud diagnóstica de ambas pruebas (GG y PCR) frente al estándar correcto.

Las falencias de la GG para el diagnóstico de la malaria gestacional en zonas endémicas resultan más graves al considerar que esta enfermedad se presenta en zonas de bajo ingresos económicos donde es difícil implementar pruebas moleculares (por su mayor costo frente a la GG). Como consecuencia, se presenta un alto subregistro de casos con GG y un alto número de gestantes infectadas sin tratamiento que pueden avanzar a efectos deletéreos en el feto, el neonato y la madre, como anemia, malaria cerebral, malaria grave, muerte, entre otros ^1^ ^,^ ^2^. Todo esto tiene mayor relevancia al considerar que, entre los objetivos de la OMS para la MAE se encuentra el control y erradicación de esta enfermedad, lo cual no se logrará mediante la tamización y diagnóstico con GG. A estas dificultades de exactitud diagnóstica se suma la escasez de recursos para lograr una prevención efectiva de la infección en gestantes, mediante estrategias clásicas como uso de mosquiteros, mitigación de condiciones de riesgo en las viviendas ^29^, acceso a medicamentos profilácticos en las zonas, entre otras ^30^ ^,^ ^31^.

A pesar de la exhaustividad del protocolo de esta revisión sistemática, se hallaron muy pocos estudios de evaluación diagnóstica de la GG, probablemente por tratarse de la prueba estándar recomendada para el diagnóstico en la mayoría de los guías internacionales. Este hallazgo también se podría sustentar en el hecho de que, en este campo de la evaluación diagnóstica para malaria, predominan los estudios que analizan las pruebas de diagnóstico rápido, las cuales están tomando fuerza al enfocarse en moléculas específicas del parásito y permitir diferenciar la especie de *Plasmodium.* En este orden de ideas, es importante remarcar que las recomendaciones técnicas del diagnóstico y seguimiento del tratamiento de la malaria en países endémicos como Colombia ^32^, sugieren el uso de GG y pruebas rápidas, a pesar de su baja sensibilidad en pacientes asintomáticas y con baja parasitemia, dadas las dificultades para implementar el diagnóstico molecular en zonas alejadas de los centros poblados. En estos contextos, se restringe el uso de pruebas moleculares a los casos sintomáticos con GG y prueba rápida negativas, ante resultados discordantes y en infecciones por especies diferentes a *P. falciparum *o *P. vivax, *como apoyo al control de calidad del programa de diagnóstico y para monitorear zonas de baja transmisión. Con base en los resultados de este metaanálisis, se muestra la importancia de ampliar el uso de pruebas moleculares para la vigilancia de la MAE donde se presenta una elevada frecuencia de infecciones asintomáticas y submicroscópicas ^10^.

La mayoría de los artículos metaanalizados usaron nPCR como estándar y muy pocos qPCR, esto podría atribuirse al alto costo y mayor complejidad en la infraestructura requerida y en los protocolos de este tipo de pruebas. Blanquicet *et al*. ^33^ refieren que, aunque ambas pruebas diagnósticas llevan a resultados confirmatorios, la qPCR sigue siendo una prueba que aporta más confianza y seguridad en el diagnóstico oportuno de MAE ya que al dar la cuantificación del parásito permite la detección y seguimiento de casos con una parasitemia inferior a la detectada por microscopía (10-30 parásitos/µL). A estas ventajas de la qPCR se suma el hecho de tener menor riesgo de contaminación, debido que la nPCR requiere al menos dos pasos y se trata de un sistema abierto, caso contrario a la qPCR que se desarrolla en termociclador cerrado ^33^ ^,^ ^34^.

En adición a lo anterior, la qPCR se denomina una subvariante de la PCR en tiempo real que a su vez es un mejoramiento de nPCR. En estas tres tipologías de PCR es posible la detección de género y especie, pero en las dos primeras la cuantificación por medio de los Ct o umbral de ciclos alcanza valores de parasitemia muy bajos como se demuestra en el estudio de Khainar *et al*. ^35^ donde se registra una mayor sensibilidad analítica de la qPCR en cuatro especies de *Plasmodium*, con límites de detección de 11 copias de ADNr/µl.

Entre las principales limitaciones de este estudio se destacan la imposibilidad de profundizar en algunas metarregresiones que permitieran evidenciar el rol de variables como la presencia de síntomas (anemia o fiebre), el número de embarazos, la captación de muestras al momento del parto, entre otras características clínicas y ginecológicas, debido a que los pocos estudios que aluden a estas características no segregaron los parámetros de exactitud acorde con estas variables, o fue muy bajo el número de los que sí lo realizaron, impidiendo la estimación de medidas combinadas. Por estas mismas razones no fue posible metaanalizar la exactitud diagnóstica de la GG para malaria placentaria y congénita. Estas limitaciones constituyen las principales líneas de trabajo para estudios posteriores en este campo.

En conclusión, mediante un protocolo exhaustivo se evidenció el bajo desarrollo de investigaciones sobre la exactitud diagnóstica de la GG en MAE. Pese a ello, esta investigación logró metaanalizar los pocos estudios disponibles, logrando un elevado número de gestantes evaluadas en quienes se demostró que la microscopía tiene un desempeño deficiente para el diagnóstico de infecciones asintomáticas o de baja parasitemia, lo que afianza la importancia de implementar otro tipo de técnicas en el seguimiento y control de las infecciones por malaria en las gestantes, con el fin de lograr el control y posible eliminación de la MAE.
